# Expression Levels of Long Non-Coding RNAs Change in Models of Altered Muscle Activity and Muscle Mass

**DOI:** 10.3390/ijms21051628

**Published:** 2020-02-27

**Authors:** Keisuke Hitachi, Masashi Nakatani, Shiori Funasaki, Ikumi Hijikata, Mizuki Maekawa, Masahiko Honda, Kunihiro Tsuchida

**Affiliations:** 1 Division for Therapies against Intractable Diseases, Institute for Comprehensive Medical Science (ICMS), Fujita Health University, Toyoake 470-1192, Japan; hkeisuke@fujita-hu.ac.jp (K.H.); nakatani@fujita-hu.ac.jp (M.N.);; 2 Department of Biochemistry, Kindai University Faculty of Medicine, Osaka-Sayama 589-8511, Japan; mhonda@med.kindai.ac.jp; 3 Department of Bioscience and Genetics, National Cerebral and Cardiovascular Center Research Institute, Suita 564-8565, Japan

**Keywords:** long non-coding RNAs, disuse atrophy, muscle wasting, muscle hypertrophy, myostatin, skeletal muscle mass

## Abstract

Skeletal muscle is a highly plastic organ that is necessary for homeostasis and health of the human body. The size of skeletal muscle changes in response to intrinsic and extrinsic stimuli. Although protein-coding RNAs including myostatin, NF-κβ, and insulin-like growth factor-1 (IGF-1), have pivotal roles in determining the skeletal muscle mass, the role of long non-coding RNAs (lncRNAs) in the regulation of skeletal muscle mass remains to be elucidated. Here, we performed expression profiling of nine skeletal muscle differentiation-related lncRNAs (*DRR*, *DUM1*, *linc-MD1*, *linc-YY1*, *LncMyod*, *Neat1*, *Myoparr*, *Malat1*, and *SRA*) and three genomic imprinting-related lncRNAs (*Gtl2*, *H19*, and *IG-DMR*) in mouse skeletal muscle. The expression levels of these lncRNAs were examined by quantitative RT-PCR in six skeletal muscle atrophy models (denervation, casting, tail suspension, dexamethasone-administration, cancer cachexia, and fasting) and two skeletal muscle hypertrophy models (mechanical overload and deficiency of the *myostatin* gene). Cluster analyses of these lncRNA expression levels were successfully used to categorize the muscle atrophy models into two sub-groups. In addition, the expression of *Gtl2*, *IG-DMR*, and *DUM1* was altered along with changes in the skeletal muscle size. The overview of the expression levels of lncRNAs in multiple muscle atrophy and hypertrophy models provides a novel insight into the role of lncRNAs in determining the skeletal muscle mass.

## 1. Introduction

Skeletal muscle is an organ that plays important roles in motion, postural maintenance, and metabolic adaptation. The adult skeletal muscle exhibits high plasticity and its mass can be altered by intrinsic and extrinsic stimuli. The skeletal muscle mass is decreased not only in diseases, such as cancer cachexia, neuromuscular disorders, chronic kidney disease, heart failure, and chronic obstructive pulmonary disease, but also as a consequence of aging, immobilization, and malnutrition [1,2,3,4,5,6,7,8]. The latter type of loss in the muscle mass is better known as muscle atrophy or wasting condition, which affects the activities of daily living and leads to increased mortality from diseases [9,10]. Activated signaling of myostatin, NF-κβ, and glucocorticoid leads to dysregulation of the ubiquitin-proteasome or autophagy-lysosome systems, inducing muscle atrophy and wasting [11,12,13]. In contrast, moderate exercise and good nutrition increase the skeletal muscle mass (known as muscle hypertrophy) through the activation of insulin-like growth factor-1 (IGF-1)/Akt/mTOR (mammalian target of rapamycin) and β-adrenergic pathways [12,14]. Epidemiological studies have shown that people with high skeletal muscle mass have a lower prevalence of various diseases and a longer life expectancy [15,16]. Thus, understanding the molecular mechanisms controlling skeletal muscle mass is important to extend the healthy life expectancy in humans.

Recent advances in techniques for nucleic acid detection have revealed the presence of numerous long non-coding RNAs (lncRNAs), which are defined as RNAs longer than 200 bases in length [17]. Although lncRNAs do not code for proteins, dosage compensation and genomic imprinting have been known to be controlled by lncRNAs [18,19,20,21]. It is now clear that lncRNAs work as transcriptional, epigenetic, and translational regulators; structural cores; and perform other functions through their interaction with several essential proteins [22,23]. During skeletal muscle formation, a lncRNA, *SRA*, is required for the terminal differentiation of myoblasts by enhancing the transcriptional activity of myoblast determination protein 1 (MyoD) [24], one of the key myogenic transcription factors. The *DRR* enhancer RNA, also known as *MUNC*, is expressed from the distal regulatory element of the *Myod1* gene, which codes for MyoD, and promotes myogenic differentiation [25,26]. *LncMyoD* is in the immediate vicinity of the *Myod1* gene and is required for terminal muscle differentiation through the translational regulation of genes involved in proliferation [27]. Other lncRNAs, such as *linc-MD1*, *DUM1*, *linc-YY1*, *Malat1*, and *Neat1*, also regulate myogenesis [28,29,30,31,32,33]. However, the roles of these lncRNAs in the regulation of skeletal muscle mass remain largely unknown.

We previously identified an lncRNA, *Myoparr*, from the promoter region of the *myogenin* gene, which codes for a transcriptional factor that is indispensable for skeletal muscle differentiation [34]. *Myoparr* interacts with a transcriptional co-activator, DEAD (Asp-Glu-Ala-Asp) box polypeptide 17 (Ddx17), and is essential both for the specification of myoblasts into the differentiation lineage and for the myoblast cell cycle withdrawal. Intriguingly, *Myoparr* is also involved in the induction of muscle atrophy caused by surgical denervation in adult skeletal muscle in mice [34,35]. These findings suggest that lncRNAs involved in muscle differentiation might also be involved in the regulation of skeletal muscle mass. In addition, we previously showed that muscle hypertrophy induced by *myostatin* deficiency increased the expression levels of genomic imprinting-related lncRNAs, *Gtl2* (also known as *Meg3*) and *IG-DMR* [36]. *Gtl2* promotes bovine myoblast differentiation by acting as an miR-135 sponge [37]. *H19*, another genomic imprinting-related lncRNA, also regulates myogenic differentiation [38,39,40], and knockout mice of *H19* show muscle hypertrophy and hyperplasia [41]. Thus, in the current study, to reveal the roles of lncRNAs in the regulation of skeletal muscle mass, we performed expression profiling of nine lncRNAs (*DRR*, *DUM1*, *linc-MD1*, *linc-YY1*, *LncMyod*, *Neat1*, *Myoparr*, *Malat1*, and *SRA*), whose roles in skeletal muscle differentiation were well established [24,25,26,27,28,29,30,31,32,33], and three genomic imprinting-related lncRNAs (*Gtl2*, *H19*, and *IG-DMR*) in six muscle atrophy and two muscle hypertrophy models in mice. By using the expression data sets of these lncRNAs, we could classify the muscle atrophy conditions into two sub-groups. Our comprehensive expression data of 12 lncRNAs provide an important insight into the roles of these lncRNAs in the regulation of skeletal muscle mass.

## 2. Results

### 2.1. Changes in the Expression of Skeletal Muscle Differentiation-Related lncRNAs during Muscle Atrophy

We examined the expression of nine skeletal muscle differentiation-related lncRNAs (*Myoparr*, *linc-MD1*, *LncMyod*, *DRR*, *DUM1*, *linc-YY1*, *Malat1*, *Neat1*, and *SRA*) in six muscle atrophy models in mice. The first three models, including denervation, casting, and tail suspension, are the disuse atrophy group. Sciatic nerve transection significantly decreased the tibialis anterior (TA) muscle weight by 18.9% compared to that in the control side that was sham operated after 7 days of surgical denervation (Table 1). A significant decrease of 9.9% in the muscle weight was also observed after 7 days of casting (Table 1). The disuse of skeletal muscle for 3 days in the tail suspension model significantly decreased the TA muscle weight by 10.9% (Table 1). The next three models are the systemically muscle wasting group. Glucocorticoids are strong inducers of systemic muscle wasting [42]. Continuous administration of a high dose of glucocorticoid (dexamethasone, Dex) for 7 days significantly decreased the weight of the TA muscles by 9.8% (Table 1). Cancer cachexia also systemically induces the loss of skeletal muscle mass and this symptom is prominent in cancer patients [43]. Tumor-bearing mice with cachexia-inducible colon-26 adenocarcinoma (C26) cells have been used as cancer cachexia models [44]. C26-transplanted mice showed a significant decrease of 22.4% in the skeletal muscle mass as compared to the phosphate-buffered saline (PBS)-administrated control mice (Table 1). In addition, by 2 days of fasting, a 14.4% decrease in the weight of TA muscles was observed compared to that for control mice fed normally (Table 1). 

**Figure 1:**
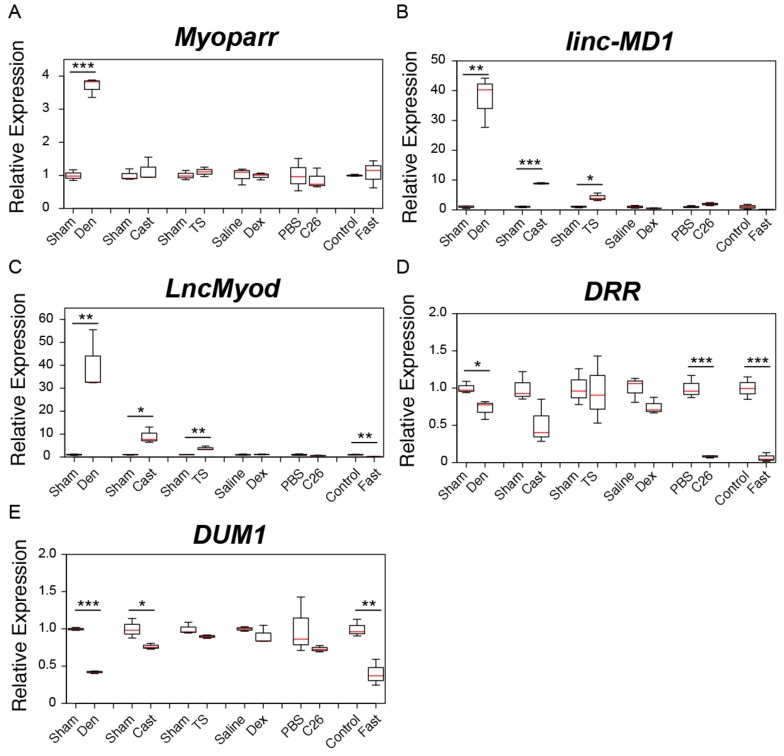
Changes in the expression of skeletal muscle differentiation-related lncRNAs in six muscle atrophy conditions. (**A**–**E**) Box-and-whisker plots showing the results of quantitative RT-PCR (qRT-PCR) for *Myoparr* (**A**), *linc-MD1* (**B**), *LncMyod* (**C**), *DRR* (**D**), and *DUM* (**E**) expression in multiple muscle atrophy models. Red lines indicate the median values. Lower and upper box limits are 25th and 75th percentiles, respectively. Whiskers indicate the maximum and minimum values. Sham; tibialis anterior (TA) muscles of sham-operated C57BL/6J mice. Den; denervated TA muscles of C57BL/6J mice. Cast; casting-operated TA muscles of C57BL/6J mice. TS; TA muscles of tail suspension-operated C57BL/6J mice. Saline; TA muscles of saline-injected C57BL/6J mice. Dex; TA muscles of dexamethasone-injected C57BL/6J mice. PBS; TA muscles of control phosphate-buffered saline (PBS)-injected CD2F1 mice. C26; TA muscles of C26 tumor-bearing CD2F1 mice. Control; TA muscles of C57BL/6J mice provided with water and food ad libitum. Fast; TA muscles of fasting C57BL/6J mice. qRT-PCR data were normalized to *Rpl26* expression and shown as relative expression. *n* = 3 per group. * *p* < 0.05. ** *p* < 0.01. *** *p* < 0.001.

We previously showed that surgical denervation increases the expression level of *Myoparr* in skeletal muscles [34]. Thus, we first examined *Myoparr* expression in adult skeletal muscles after surgical denervation. In accordance with a previous report [34], *Myoparr* expression was significantly increased by denervation (Figure 1A). However, the expression level of *Myoparr* was not changed significantly in other muscle atrophy models (Figure 1A). Intriguingly, we found that surgical denervation significantly increased the expression level of *linc-MD1* in comparison to the expression in the muscle on the control side (Figure 1B). These results are consistent with our previous findings that *Myoparr* activates the expression levels of *miR-133b* and *miR-206*, which are located on the *linc-MD1* locus during myogenic differentiation [34]. The expression level of *linc-MD1* was also significantly increased after the casting and tail suspension treatments but did not change significantly either by the Dex treatment, cancer cachexia, or in the fasting mice (Figure 1B). In addition to *linc-MD1*, the denervation, casting, and tail suspension treatments significantly increased the expression level of *LncMyod* (Figure 1C). On the other hand, the expression level of *LncMyod* was observed to decrease in the fasting mice (Figure 1C). Although not significant in all muscle atrophy models, we observed that the expression levels of *DRR* and *DUM1* tended to be decreased in all models (Figure 1D and 1E). The expression levels of *linc-YY1*, *Malat1*, *Neat1*, and *SRA* showed significant changes in each model, but they did not show consistent changes across the different muscle atrophy models (Supplementary Figure S1A–D).

### 2.2. Changes in the Expression of Genomic Imprinting-Related lncRNAs during Muscle Atrophy

**Figure 2:**
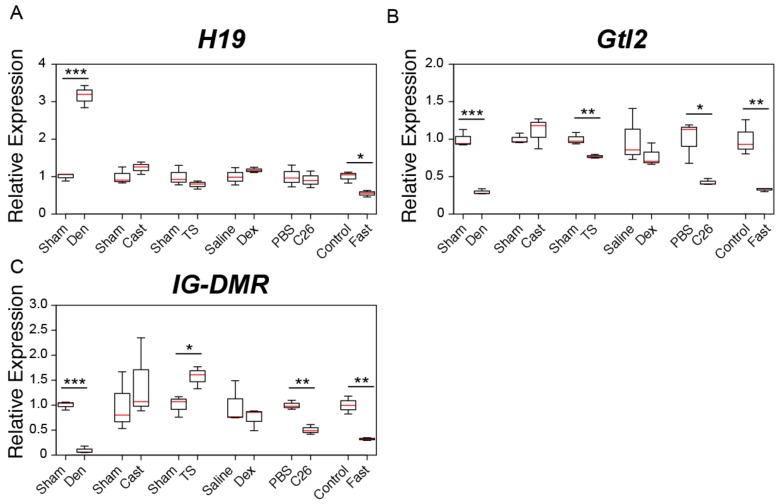
Changes in the expression of genomic imprinting-related lncRNAs in six muscle atrophy conditions. (**A**–**C**) Box-and-whisker plots showing the results of quantitative RT-PCR (qRT-PCR) for *H19* (**A**), *Gtl2* (**B**) and *IG-DMR* (**C**) expression in multiple muscle atrophy models. Sham; tibialis anterior (TA) muscles of sham-operated C57BL/6J mice. Den; denervated TA muscles of C57BL/6J mice. Cast; casting-operated TA muscles of C57BL/6J mice. TS; TA muscles of tail suspension-operated C57BL/6J mice. Saline; TA muscles of saline-injected C57BL/6J mice. Dex; TA muscles of dexamethasone-injected C57BL/6J mice. PBS; TA muscles of control phosphate-buffered saline (PBS)-injected CD2F1 mice. C26; TA muscles of C26 tumor-bearing CD2F1 mice. Control; TA muscles of C57BL/6J mice provided with water and food ad libitum. Fast; TA muscles of fasting C57BL/6J mice. qRT-PCR data were normalized to *Rpl26* expression and shown as relative expression. *n* = 3 per group. * *p* < 0.05. ** *p* < 0.01. *** *p* < 0.001.

We next examined the expression of three genomic imprinting-related lncRNAs (*H19*, *Gtl2*, and *IG-DMR*) in six muscle atrophy models. Intriguingly, we found increased *H19* expression after 7 days of surgical denervation (Figure 2A). As in the case of *linc-MD1*, this result is consistent with *Myoparr* activating *H19* expression during myogenic differentiation [34]. Fasting also significantly altered the expression level of *H19*, but its expression was not changed significantly in the other four muscle atrophy models (Figure 2A). We observed significant decreases in *Gtl2* expression by the denervation, tail suspension, and cancer cachexia and in the fasting mice (Figure 2B). The *IG-DMR* expression was also significantly decreased by the denervation and cancer cachexia and in the fasting mice, but the expression level of *IG-DMR* was observed to significantly increase by the tail suspension treatment (Figure 2C).

### 2.3. Cluster Analysis of Changes in the Expression of lncRNAs in Muscle Atrophy Models

**Figure 3:**
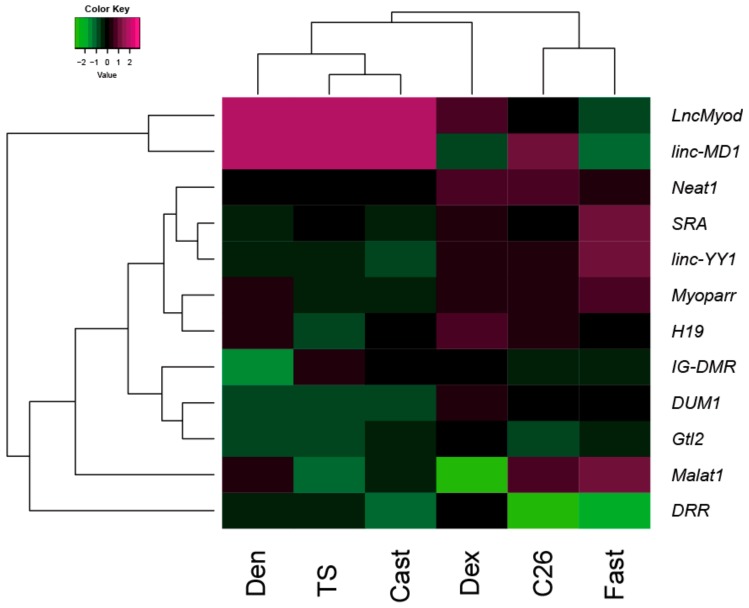
Cluster analysis of the expression profiling of lncRNAs in muscle atrophy conditions. The result of the cluster analysis of the expression profile of lncRNAs in muscle atrophy is shown as a heatmap (values are z-scores). Den; denervation treatment. TS; tail suspension treatment. Cast; casting treatment. Dex; dexamethasone administration. C26; cancer cachexia. Fast; fasting treatment.

We classified the muscle atrophy models using the expression profiling of 12 lncRNAs. The value of the log2 fold change in the expression levels of lncRNAs in each model was converted to a z-score and cluster analysis was performed. The result is shown as a heatmap sorted by hierarchical clustering trees. We could classify the six muscle atrophy models into two sub-groups; one was the disuse atrophy group containing denervation, tail suspension, and casting treatments. The other was the systemically wasting group containing dexamethasone administration, C26-induced cachexia, and fasting (Figure 3). A subset of lncRNAs showed similar expression profiles in each group. Intriguingly, highly upregulated expression levels of *LncMyod* and *linc-MD1* were observed in the disuse group but not in the systemically wasting group.

### 2.4. Changes in the Expression Levels of Skeletal Muscle Differentiation-Related lncRNAs in Skeletal Muscle Hypertrophy Conditions

Altered lncRNA expression levels in muscle atrophy models prompted us to examine the expression profiling of nine skeletal muscle differentiation-related lncRNAs (*Myoparr*, *linc-MD1*, *LncMyod*, *DRR*, *DUM1*, *linc-YY1*, *Malat1*, *Neat1*, and *SRA*) in the skeletal muscle hypertrophy condition. Because of the difficulty in inducing muscle hypertrophy in mice by resistance training, we used both genetic hypertrophy and mechanical overload (MOV)-induced chronic hypertrophy models for the analysis. As a genetic hypertrophy model, we used *myostatin* knockout mice [45]. Loss of the *myostatin* gene in mice resulted in a significant increase of 81.5% in the TA muscle mass compared to that in the wild-type mice (Table 1). Mechanical overload of the plantaris muscle by synergistic ablation was achieved by surgically removing the soleus and gastrocnemius muscles. Although not completely identical, plantaris muscle has a similar fiber-type composition with that of TA muscle in mice [46]. To examine the effect of chronic muscle hypertrophy as well as *myostatin* knockout mice, we collected muscles 6 weeks after surgery in the MOV model. By 6 weeks of synergistic ablation, an 85.7% increase in the weight of plantaris muscles was observed compared to that in sham-operated mice (Table 1).

**Figure 4:**
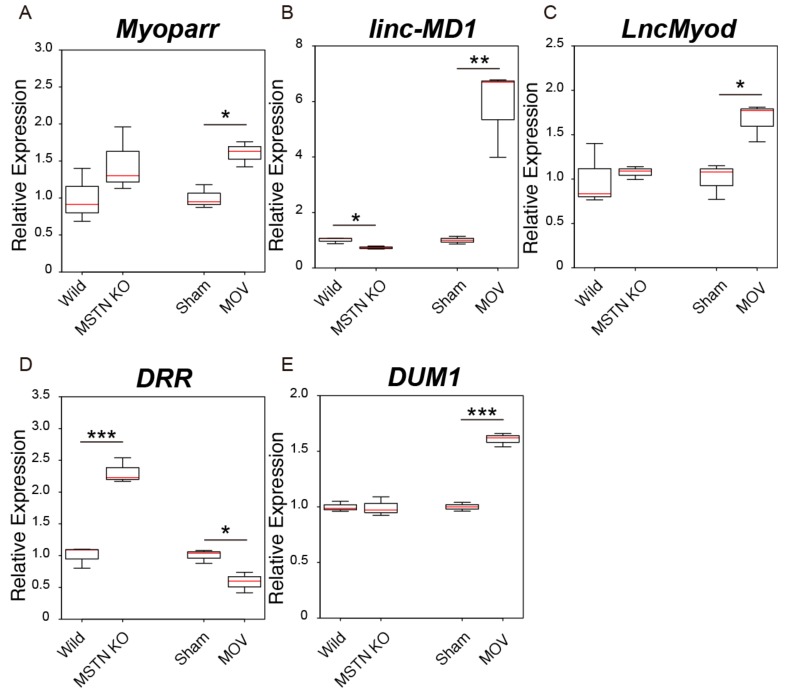
Changes in the expression levels of skeletal muscle differentiation-related lncRNAs in skeletal muscle hypertrophy conditions. (**A**–**E**) Box-and-whisker plots showing the results of quantitative RT-PCR (qRT-PCR) for *Myoparr* (**A**), *linc-MD1* (**B**), *LncMyod* (**C**), *DRR* (**D**), and *DUM1* (**E**) expression in two muscle hypertrophy conditions. Wild; tibialis anterior (TA) muscles of control C57BL/6J mice. MSTN KO; TA muscles of C57BL/6J-background *myostatin* knockout mice. Sham; plantaris muscles of sham-operated C57BL/6J mice. MOV; plantaris muscles of mechanical overload (MOV)-operated C57BL/6J mice. qRT-PCR data were normalized to *Rpl26* expression and shown as relative expression. *n* = 3 per group. * *p* < 0.05. ** *p* < 0.01. *** *p* < 0.001.

Although the expression level of *Myoparr* was not changed significantly by the *myostatin* deficiency, the MOV treatment significantly increased the expression level of *Myoparr* (Figure 4A). The expression level of *linc-MD1* was significantly decreased and increased by the *myostatin* deficiency and the MOV treatment, respectively (Figure 4B). A significant increase in *LncMyod* expression was only observed by the MOV treatment (Figure 4C). The expression level of *DRR* was highly altered by both the *myostatin* deficiency and the MOV treatment, but the direction of the changes was the opposite in both models (Figure 4D). The expression level of *DUM1* was significantly increased only by the MOV treatment (Figure 4E). Although the administration of recombinant myostatin decreases the expression of *Malat1*, both in vitro and in vivo [47], no significant increase or decrease in the expression of other skeletal muscle differentiation-related lncRNAs, including *Malat1*, was found in *myostatin* knockout mice (Supplementary Figure S2A–D). The MOV treatment also affected the expression levels of *linc-YY1* and *SRA* (Supplementary Figure S2A,D).

### 2.5. Changes in the Expression Levels of Genomic Imprinting-Related lncRNAs in Skeletal Muscle Hypertrophy Conditions

**Figure 5:**
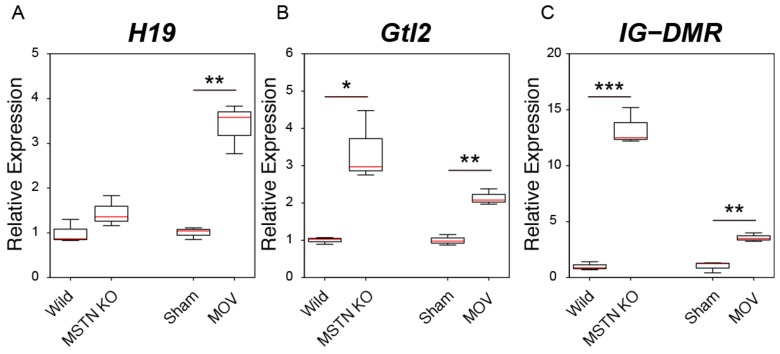
Changes in the expression levels of genomic imprinting-related lncRNAs in skeletal muscle hypertrophy conditions. (**A**–**C**) Box-and-whisker plots showing the results of quantitative RT-PCR (qRT-PCR) for *H19* (**A**), *Gtl2* (**B**), and *IG-DMR* (**C**) expression in two muscle hypertrophy conditions. Wild; TA muscles of control C57BL/6J mice. MSTN KO; TA muscles of C57BL/6J-background *myostatin* knockout mice. Sham; plantaris muscles of sham-operated C57BL/6J mice. MOV; plantaris muscles of mechanical overload (MOV)-operated C57BL/6J mice. qRT-PCR data were normalized to *Rpl26* expression and shown as relative expression. *n* = 3 per group. * *p* < 0.05. ** *p* < 0.01. *** *p* < 0.001.

We next examined the expression of three genomic imprinting-related lncRNAs (*H19*, *Gtl2*, and *IG-DMR*) in the muscle hypertrophy condition. The expression level of *H19* was significantly increased by the MOV treatment but not by the *myostatin* deficiency (Figure 5A). In accordance with the results of our previous study [36], *myostatin* deficiency significantly increased the expression levels of *Gtl2* and *IG-DMR* (Figure 5B,C). As well as in the case of *myostatin* deficiency, significant increases in the expression levels of *Gtl2* and *IG-DMR* were also observed after the MOV treatment (Figure 5B,C).

**Figure 6:**
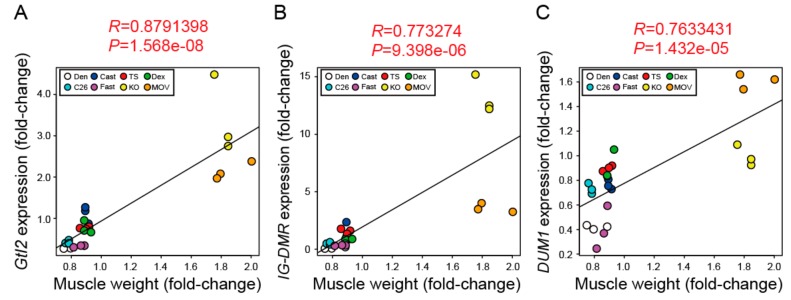
The expression levels of *Gtl2*, *IG-DMR*, and *DUM1* show a positive correlation with skeletal muscle weight. (**A**–**C**) Pearson correlation plot with a coefficient of correlation (*R*) and *p*-value (*P*) between skeletal muscle weight (fold-change with respect to control) and *Gtl2* (**A**), *IG-DMR* (**B**), and *DUM1* (**C**) expression (fold-change with respect to control) in multiple muscle atrophy and hypertrophy models. Each dot indicates the independent samples (*n* = 3 per group). Dots with white, blue, red, green, light blue, purple, yellow, and orange indicate the denervated tibialis anterior (TA) muscles of C57BL/6J mice (Den), the casting-operated TA muscles of C57BL/6J mice (Cast), the TA muscles of tail suspension-operated C57BL/6J mice (TS), the TA muscles of dexamethasone-injected C57BL/6J mice (Dex), the TA muscles of C26 tumor-bearing CD2F1 mice (C26), the TA muscles of fasting C57BL/6J mice (Fast), the TA muscles of C57BL/6J-background *myostatin* knockout mice (KO), and the plantaris muscles of mechanical overload (MOV)-operated C57BL/6J mice (MOV), respectively.

We finally performed a correlation analysis between the muscle weight and expression levels of lncRNAs in multiple muscle atrophy and hypertrophy models and found that the changes in skeletal muscle mass showed a positive correlation with the expression levels of *Gtl2* and *IG-DMR* (*R* = 0.88 and 0.77, respectively, Figure 6A,B). Although no significant increase in *DUM1* expression was observed in *myostatin* knockout mice, *DUM1* expression also showed a positive correlation with the changes in skeletal muscle mass (*R* = 0.76, Figure 6C). Thus, these results indicate that the expression levels of *Gtl2*, *IG-DMR*, and *DUM1* are highly correlated with the skeletal muscle mass.

## 3. Discussion

Recent findings indicate that denervation, immobilization, cancer cachexia, chronic kidney disease, fasting, and aging affect the expression of *Pvt1*, *lncMUMA*, *Atrolnc-1*, and *MAR1* lncRNAs in the skeletal muscles [48,49,50,51]. In addition, the expression of *Myoparr* and *Chronos* is increased by surgical denervation and aging, respectively, and regulates skeletal muscle mass through BMP signaling [35,52], which antagonizes muscle atrophy [53,54]. Other lncRNAs, namely *AK017368*, *Charme*, and *lnc-mg*, are also involved in the regulation of skeletal muscle mass through multiple mechanisms [55,56,57]. Therefore, lncRNAs have been thought to be new regulators controlling the skeletal muscle mass. Although the pivotal roles of lncRNAs (*DRR*, *DUM1*, *linc-MD1*, *linc-YY1*, *LncMyod*, *Neat1*, *Malat1*, and *SRA*) in skeletal muscle differentiation have been reported [24,25,26,27,28,29,30,31,32,33], their involvement in the regulation of skeletal muscle mass is largely unknown. In this study, we performed expression profiling of nine lncRNAs, whose expression was related to skeletal muscle differentiation, and three lncRNAs involved in genomic imprinting, using skeletal muscle atrophy and hypertrophy models. Comprehensive analysis of the expression of lncRNAs showed no common changes in the expression during muscle atrophy and hypertrophy situations, suggesting that the expression of these lncRNAs is complex and is tightly regulated during various muscle atrophy and hypertrophy conditions. However, we report the following new findings: (1) Muscle atrophy models can be divided into two sub-groups based on the expression of lncRNAs, especially the expression levels of *linc-MD1* and *LncMyod*; and (2) the expression levels of *Gtl2*, *IG-DMR*, and *DUM1* are highly correlated with skeletal muscle mass. Collectively, these findings suggest that the lncRNAs involved in muscle differentiation and genomic imprinting might also be involved in the regulation of skeletal muscle mass.

Our cluster analysis of comprehensive expression profiling of lncRNAs classified the six muscle atrophy models into two sub-groups, namely the disuse-mediated atrophy and systemically wasting atrophy sub-groups. This classification was mainly dependent on the expression of *linc-MD1* and *LncMyod*; the expression levels of *linc-MD1* and *LncMyod* were largely increased in the disuse models, including the denervation, casting, and tail suspension models, but not in the systemically wasting models, including the dexamethasone-administration, cancer cachexia, and fasting models. During muscle differentiation, the expression of *linc-MD1* and *LncMyod* is controlled by the MyoD protein [27,28]. Although denervation and immobilization activate *Myod1* expression [58,59], considering the fact that immobilization did not increase the expression of *DUM1*, *linc-YY1*, *Myoparr*, and *H19*, whose expression levels are also positively controlled by the MyoD protein during muscle differentiation [29,30,34,60], molecular mechanisms other than those involving MyoD might regulate the expression of *linc-MD1* and *LncMyod* in the disuse-mediated muscle atrophy conditions. Denervation and MOV treatments also increased *Myoparr* expression; however, it remains unclear how these treatments increased the expression level of *Myoparr*. Therefore, future studies investigating factors whose expression changes in common in denervation and MOV models may reveal the molecular mechanism regulating *Myoparr* expression in adult skeletal muscle. Intriguingly, *linc-MD1* increases the myocyte-specific enhancer factor 2C (MEF2C) and mastermind-like protein 1 (MAML1) expression by sponging *miR-133* and *miR-135* during skeletal muscle differentiation [28]. Baruffaldi et al. showed that MEF2C promotes the growth of myofibers by activating Akt/mTOR/S6K signaling [61]. MAML1 promotes the transcriptional activity of MEF2C [62]. Therefore, the increased *linc-MD1* expression would work as a compensatory mechanism, attenuating the muscle atrophy in the disuse conditions by activating MEF2C. By contrast, despite the fact that IGF2-mRNA-binding protein 2 (IMP2) is required to maintain the skeletal muscle mass [63], *LncMyoD* blocks the function of IMP2 in muscle differentiation [27], suggesting that increased *LncMyoD* expression might contribute to the cause of muscle atrophy in the disuse situations. The expression levels of *linc-MD1* and *LncMyod* were also increased by MOV treatment. Therefore, in that case, *linc-MD1* might contribute to inducing muscle hypertrophy and *LncMyod* might work as a compensatory mechanism to attenuate muscle hypertrophy, as opposed to the disuse situations. Both *linc-MD1* and *LncMyoD* are not expressed in intact mature muscle [27,28]. The mechanism by which these lncRNAs are expressed in individual muscle components, such as mature myofibers, satellite cells (adult skeletal muscle stem cells), or mesenchymal progenitor cells, during muscle atrophy conditions is unclear. Therefore, further studies examining the exact expression sites of *linc-MD1* and *LncMyoD* in multiple muscle atrophy models would be useful to reveal the roles of these lncRNAs in adult skeletal muscle.

Genomic imprinting-related lncRNAs, *Gtl2* and *IG-DMR*, are located in the *Dlk1*-*Dio3* imprinting locus at chromosome 12qF1 in mice. A paternally inherited DNA mutation of this locus, called the callipyge mutation, results in a 30%–40% increase in the hindlimb muscle mass in sheep through activation of the *Dlk1* or *Rtl1* gene [64]. On the other hand, a maternally inherited mutation of this locus increases the expression of microRNAs that inhibits *Dlk1* expression and counteracts muscle hypertrophy [65,66]. *Gtl2* is also maternally expressed from this locus and promotes muscle differentiation by sponging *miR-135* in bovine myoblasts [37]. Skeletal muscle defects were observed in mice with the maternal deletion of *Gtl2* [67]. *IG-DMR* is a maternal lncRNA and works like an enhancer RNA that controls gene expression on the maternal chromosome at the *Dlk1*-*Dio3* locus in embryonic stem cells [68]. However, it remains unclear whether *Gtl2* and *IG-DMR* are involved in the regulation of adult skeletal muscle mass. Intriguingly, our data showed that the expression levels of *Gtl2* and *IG-DMR* were increased in two muscle hypertrophy models and were decreased in multiple muscle atrophy models. We and others previously found that the expression levels of lncRNAs at this locus are gradually decreased during muscle growth and aging in mice and pigs [36,69,70,71]. It is of note that the level of *DUM1* expression, which was correlated with the skeletal muscle mass in this study, also gradually decreased during muscle growth and aging in mice [29]. Therefore, although the function of these lncRNAs in the regulation of skeletal muscle mass remains unknown, it is likely that their expression levels are useful as a promising indicator for the skeletal muscle mass.

Besides muscle mass, all of the models used in this study affect several muscle properties. For example, the disuse atrophy (denervation, casting, and tail suspension treatments) and the *myostatin* deficiency cause a slow-to-fast fiber-type shift [72,73], whereas the systemically muscle wasting (dexamethasone administration, cancer cachexia, and fasting treatments) and MOV treatment lead a fast-to-slow fiber-type shift [72,74]. Moreover, the effect of atrophy and hypertrophy models on the satellite cells is more complex. Denervation and cancer cachexia treatments activate the satellite cells [75,76]. Hindlimb suspension and fasting treatments impair satellite cell proliferation [77,78]. Immobilization decreases the number of satellite cells, whereas it increases the markers of satellite cell activation [79]. Satellite cells play little or no role in muscle hypertrophy induced by *myostatin* deficiency [80]. Expression levels of oxidative metabolic genes and extracellular matrix components are also affected in these models [81,82,83]. In addition, the contralateral sham-operated muscles, which are commonly used for the experimental controls in denervation and casting studies [84,85,86,87,88], might undergo hypertrophy to compensate for the immobilized muscles [89]. The proteasome system is also activated in the contralateral-innervated muscles compared with the muscles of non-operated mice [90]. It is also worthwhile to note that relatively low *n* numbers in each group would result in an increased false-positive change in lncRNA expression. Thus, it cannot be ruled out that the expression levels of lncRNA in our models might be influenced by the changes in these muscle properties and microenvironment.

## 4. Materials and Methods

### 4.1. Animals

The C57BL/6J and CD2F1 mice were purchased from the Japan SLC and Charles River Laboratories Japan, respectively. Mice were housed in cages with a constant temperature (24 °C) and a 12-h light:12-h dark cycle and were provided with water and food ad libitum, except during the fasting experiments. Animal experiments were conducted under protocols approved by the Institutional Animal Care and Use Committee of Fujita Health University, Japan (#AP16055, approved on June 24th, 2016). Mechanical overload experiments were approved by the Animal Care and Use Committee of the National Cerebral and Cardiovascular Center in Japan (#16035, approved on March 28th 2016) and were conducted under institutional and national guidelines. All animal experiments were performed in accordance with the ethical standards laid down in the 1964 declaration of Helsinki and its later amendments.

### 4.2. Cell Culture

Cachexia-inducible colon-26 adenocarcinoma (C26) clone 20, described previously [44], was a kind gift from Dr. K. Soda, Jichi Medical University in Japan. C26 cells were maintained in Roswell Park Memorial Institute (RPMI) 1640 medium (Wako, Osaka, Japan), supplemented with 10% fetal bovine serum (HyClone, GE Healthcare, Little Chalfont, Buckinghamshire, UK), 2 mM l-glutamine (Thermo Fisher Scientific, Waltham, MA, USA), and PS (100 units of penicillin G per mL, 10 μg of streptomycin sulphate per mL, Thermo Fisher Scientific) in an atmosphere of 5% CO_2_ at 37 °C.

### 4.3. Muscle Atrophy and Hypertrophy Models

For induction of muscle atrophy by denervation, a 3-mm segment of the sciatic nerve was excised from the hind leg of each 8-week-old male C57BL/6J mouse under anesthesia, as described previously [34]. Contralateral non-denervated hindlimb served as the control. Seven days after the operation, the mice were sacrificed and the TA muscles were collected, measured, and used for RNA preparation.

Left hindlimb of 8-week-old male C57BL/6J mice was immobilized in plaster casts under anesthesia, as described previously, for the induction of muscle atrophy by casting [91]. The right hindlimb was allowed to move freely and was used as the control. Mice were sacrificed seven days later, and the TA muscles were collected, measured, and used for RNA preparation.

Muscle atrophy was induced by tail suspension as follows: 8-week-old male C57BL/6J mice were suspended for 3 days by their tails, as described previously, such that the hindlimbs could not touch the floor, wall, and the lid of the cage [92]. Three days later, the mice were sacrificed. The TA muscles were then collected, measured, and used for RNA preparation.

Muscle atrophy was also induced by dexamethasone as follows: 7-week-old male C57BL/6J mice were intraperitoneally administrated 25 mg/kg/day water-soluble dexamethasone (Sigma-Aldrich, St. Louis, MO, USA) for 7 days under anesthesia, as described previously [93,94]. Mice administered normal saline were used as controls. The mice were sacrificed, and the TA muscles were collected, measured, and used for RNA preparation after dexamethasone treatment.

Trypsinized C26 cells were washed with PBS and 1 × 10^6^ cells resuspended in 200 µL of PBS were subcutaneously injected into the dorsum of 6-week-old male CD2F1 mice under anesthesia, as described previously, to induce muscle atrophy by cachexia [44]. Equal volumes of PBS were injected into age-matched mice in the control group. The mice were sacrificed, and the TA muscles were collected, measured, and used for RNA preparation 14 days after the injection.

For the induction of muscle atrophy by fasting, 8-week-old male C57BL/6J mice were allowed free access to water but were prohibited from eating solid food for 2 days, as described previously [95]. Control mice were provided water and food ad libitum. Next, the mice were sacrificed, following which the TA muscles were collected, measured, and used for RNA preparation.

*Myostatin* knockout mice, described previously [45], were a kind gift from Dr. S.-J. Lee, Johns Hopkins University (Baltimore, MD, USA). Age-matched C57BL/6J mice purchased from the Japan SLC were used as controls [36]. The TA muscles were collected from 9-week-old male mice under anesthesia. After measuring the weight, the TA muscles were used for RNA preparation.

For muscle hypertrophy model induction using MOV, the soleus and gastrocnemius muscles were surgically removed from 10-week-old male C57BL/6J mice under anesthesia, as described previously [74]. Sham-operated mice were used as controls. Six weeks after the surgery, mice were sacrificed and then the plantaris muscles were collected, measured, and used for RNA preparation.

### 4.4. RNA Purification, Reverse Transcription Reaction, and Quantitative PCR

Total RNA was extracted from the TA or plantaris muscles and purified using the miRNeasy Mini Kit (QIAGEN, Hilden, Germany) with DNase I (QIAGEN) treatment, according to the manufacturer’s protocol. One microgram total RNA was subjected to the reverse transcription reaction using SuperScript III reverse transcriptase (Thermo Fisher Scientific) or Protoscript II reverse transcriptase (New England Biolabs, Ipswich, MA, USA) with random primers. The quantitative real-time PCR (qRT-PCR) was conducted using SYBR Premix Ex Taq (Takara, Shiga, Japan). Data were normalized to *Rpl26* expression and shown as relative expression compared to the control samples. The raw data of qRT-PCR are shown in Supplementary Table S1. The GenBank accession numbers of lncRNAs are as follows: *Myoparr* (NR_160520), *linc-MD1* (NR_131249), *DUM1* (NR_028300), *linc-YY1* (AK081464), *Malat1* (NR_002847), *Neat1* (NR_131212), *SRA* (NM_025291), *H19* (NR_130973), and *Gtl2* (NR_027652). The full-length sequences of *DRR* (*MUNC*) and *LncMyoD* were described in [26,27], respectively. Since the full-length sequence of *IG-DMR* lncRNA has not identified yet, we used the previously described primer-pairs [68] to detect *IG-DMR* lncRNA. The primers used are listed in Table 2.

### 4.5. Statistical Analysis

Statistical analyses were performed using unpaired two-tailed Student’s *t*-tests. A value of *p* < 0.05 was considered statistically significant. Data are presented as box-and-whisker plots and statistical significance is reported in the figure legends as * *p* < 0.05, ** *p* < 0.01, *** *p* < 0.001.

### 4.6. Cluster and Correlation Analysis

The value of the log2 fold change in the expression levels of lncRNAs was converted to a z-score using R software with the Genefilter package (https://bioconductor.org/packages/release/bioc/html/genefilter.html). The z-score was used for the cluster analysis of gene expression changes under muscle atrophy. Correlation analysis was performed by R software using Pearson correlation coefficient. A value of *R* > 0.7 was considered a strong correlation.

## 5. Conclusions

The expression profiling of 12 lncRNAs, which are related to skeletal muscle differentiation and genomic imprinting, in multiple muscle atrophy and hypertrophy conditions performed in this study provides a novel insight about lncRNAs in determining the skeletal muscle mass. However, further studies including expression analysis of lncRNAs using more suitable models or animal experiments using conditional transgenic or knockout mice of these lncRNAs are needed to mechanistically relate the changes in skeletal muscle mass to lncRNA expression.

## Figures and Tables

**Table 1:** Muscle weight changes in each model.

Condition	Muscle Weight of Control Group (mg)	Muscle Weight of Treated Group (mg)	*p* Value	Strain	Muscle
**Denervation**	44.0 ± 1.0	35.7 ± 3.1	< 0.05	C57BL/6J	TA
**Casting**	47.0 ± 1.0	42.3 ± 0.6	< 0.01	C57BL/6J	TA
**Tail suspension**	49.0 ± 1.0	43.7 ± 1.5	< 0.01	C57BL/6J	TA
**Glucocorticoid administration**	44.0 ± 1.0	39.7 ± 1.2	< 0.01	C57BL/6J	TA
**Cancer cachexia**	44.7 ± 1.5	34.7 ± 0.6	< 0.001	CD2F1	TA
**Fasting**	41.7 ± 1.2	35.7 ± 1.5	< 0.01	C57BL/6J	TA
***Myostatin*** **deficiency**	43.3 ± 4.9	78.7 ± 2.3	< 0.001	C57BL/6J	TA
**Mechanical overload**	21.2 ± 0.9	39.3 ± 2.7	< 0.001	C57BL/6J	Plantaris

Data are presented as means ± SD. *n* = 3 per group. TA as abbreviation means tibialis anterior muscle.

**Table 2:** Primer sequences used for quantitative RT-PCR.

Target Name	Forward	Reverse
*Myoparr*	GTGCCCTATCGTCCATGGAG	CACTGACTTCACCTGACCCC
*linc-MD1*	GCAAGAAAACCACAGAGGAGG	GTGAAGTCCTTGGAGTTTGAG
*LncMyoD*	CTGAAGGACACAAGGTGGCTT	AACTGAGGCTCCCAGTAAGA
*DRR (MUNC)*	ACTAGATTTGCACAAGTGGTTTGA	TGTCGTTAGTAATGATTTCGATGG
*DUM1*	GGGATGCGAGTCTCCTCTTG	GACGATCATTCGCTTGACTTTG
*linc-YY1*	AGTTACAGGGAAGTTTGGGCTAC	AGGCAAAGGACGGCTGTGAG
*Malat1*	CATGGCGGAATTGCTGGTA	CGTGCCAACAGCATAGCAGTA
*Neat1*	TTGGGACAGTGGACGTGTGG	TCAAGTGCCAGCAGACAGCA
*SRA*	TCCACCTCCTTCAAGTAAGGCT	GACCTCAGTCACATGGTCAACC
*H19*	CTGCTCCAAGGTGAAGCTGA	TAGAGGCTTGGCTCCAGGAT
*Gtl2(Meg3)*	TTGCACATTTCCTGTGGGAC	AAGCACCATGAGCCACTAGG
*IG-DMR*	AGAAGCTGTGGTGGGATTGCT	AGGGCCACTTGCATCAGAAT
*Rpl26*	GGTCTATGCCCATTCGGAAGG	TCGTTCGATGTAGATGACGTACT

## Supplementary Materials

Supplementary Materials can be found at https://www.mdpi.com/1422-0067/21/5/1628/s1. Supplementary Table S1: The raw data of qRT-PCR. Figure S1: Changes in the expression of skeletal muscle differentiation-related lncRNAs in six muscle atrophy conditions. Figure S2: Changes in the expression levels of skeletal muscle differentiation-related lncRNAs in skeletal muscle hypertrophy conditions.

## References

[1] Rohm M., Zeigerer A., Machado J., Herzig S. (2019). Energy metabolism in cachexia. EMBO Rep..

[2] Casas C., Manzano R., Vaz R., Osta R., Brites D. (2016). Synaptic failure: Focus in an integrative view of ALS. Brain Plast..

[3] Oliveira E.A., Cheung W.W., Toma K.G., Mak R.H. (2018). Muscle wasting in chronic kidney disease. Pediatr. Nephrol..

[4] Suzuki T., Palus S., Springer J. (2018). Skeletal muscle wasting in chronic heart failure. ESC Heart Fail..

[5] Barreiro E., Jaitovich A. (2018). Muscle atrophy in chronic obstructive pulmonary disease: Molecular basis and potential therapeutic targets. J. Thorac. Dis..

[6] Larsson L., Degens H., Li M., Salviati L., Lee Y.I., Thompson W., Kirkland J.L., Sandri M. (2019). Sarcopenia: Aging-related loss of muscle mass and function. Physiol. Rev..

[7] Gao Y., Arfat Y., Wang H., Goswami N. (2018). Muscle atrophy induced by mechanical unloading: Mechanisms and potential countermeasures. Front. Physiol..

[8] Picot J., Hartwell D., Harris P., Mendes D., Clegg A.J., Takeda A. (2012). The effectiveness of interventions to treat severe acute malnutrition in young children: A systematic review. Health Technol. Assess..

[9] Egerman M.A., Glass D.J. (2014). Signaling pathways controlling skeletal muscle mass. Crit. Rev. Biochem. Mol. Biol..

[10] Cohen S., Nathan J.A., Goldberg A.L. (2015). Muscle wasting in disease: Molecular mechanisms and promising therapies. Nat. Rev. Drug Discov..

[11] Rodriguez J., Vernus B., Chelh I., Cassar-Malek I., Gabillard J.C., Hadj Sassi A., Seiliez I., Picard B., Bonnieu A. (2014). Myostatin and the skeletal muscle atrophy and hypertrophy signaling pathways. Cell. Mol. Life Sci..

[12] Schiaffino S., Dyar K.A., Ciciliot S., Blaauw B., Sandri M. (2013). Mechanisms regulating skeletal muscle growth and atrophy. FEBS J..

[13] Braun T.P., Marks D.L. (2015). The regulation of muscle mass by endogenous glucocorticoids. Front. Physiol..

[14] Hitachi K., Tsuchida K. (2014). Role of microRNAs in skeletal muscle hypertrophy. Front. Physiol..

[15] Heitmann B.L., Frederiksen P. (2009). Thigh circumference and risk of heart disease and premature death: Prospective cohort study. BMJ.

[16] Srikanthan P., Karlamangla A.S. (2014). Muscle mass index as a predictor of longevity in older adults. Am. J. Med..

[17] Hon C.C., Ramilowski J.A., Harshbarger J., Bertin N., Rackham O.J.L., Gough J., Denisenko E., Schmeier S., Poulsen T.M., Severin J. (2017). An atlas of human long non-coding RNAs with accurate 5’ ends. Nature.

[18] Marahrens Y., Panning B., Dausman J., Strauss W., Jaenisch R. (1997). *Xist*-deficient mice are defective in dosage compensation but not spermatogenesis. Genes Dev..

[19] Lee J.T., Davidow L.S., Warshawsky D. (1999). *Tsix*, a gene antisense to *Xist* at the X-inactivation centre. Nat. Genet..

[20] Bartolomei M.S., Zemel S., Tilghman S.M. (1991). Parental imprinting of the mouse H19 gene. Nature.

[21] Feil R., Walter J., Allen N.D., Reik W. (1994). Developmental control of allelic methylation in the imprinted mouse *Igf2* and *H19* genes. Development.

[22] Hirose T., Mishima Y., Tomari Y. (2014). Elements and machinery of non-coding RNAs: Toward their taxonomy. EMBO Rep..

[23] Quinn J.J., Chang H.Y. (2016). Unique features of long non-coding RNA biogenesis and function. Nat. Rev. Genet..

[24] Caretti G., Schiltz R.L., Dilworth F.J., Di Padova M., Zhao P., Ogryzko V., Fuller-Pace F.V., Hoffman E.P., Tapscott S.J., Sartorelli V. (2006). The RNA helicases p68/p72 and the noncoding RNA SRA are coregulators of MyoD and skeletal muscle differentiation. Dev. Cell.

[25] Mousavi K., Zare H., Dell’orso S., Grontved L., Gutierrez-Cruz G., Derfoul A., Hager G.L., Sartorelli V. (2013). eRNAs promote transcription by establishing chromatin accessibility at defined genomic loci. Mol. Cell.

[26] Mueller A.C., Cichewicz M.A., Dey B.K., Layer R., Reon B.J., Gagan J.R., Dutta A. (2015). MUNC, a long noncoding RNA that facilitates the function of MyoD in skeletal myogenesis. Mol. Cell. Biol..

[27] Gong C., Li Z., Ramanujan K., Clay I., Zhang Y., Lemire-Brachat S., Glass D.J. (2015). A long non-coding RNA, *LncMyoD*, regulates skeletal muscle differentiation by blocking IMP2-mediated mRNA translation. Dev. Cell.

[28] Cesana M., Cacchiarelli D., Legnini I., Santini T., Sthandier O., Chinappi M., Tramontano A., Bozzoni I. (2011). A long noncoding RNA controls muscle differentiation by functioning as a competing endogenous RNA. Cell.

[29] Wang L., Zhao Y., Bao X., Zhu X., Kwok Y.K.Y., Sun K., Chen X., Huang Y., Jauch R., Esteban M.A. (2015). LncRNA *Dum* interacts with Dnmts to regulate *Dppa2* expression during myogenic differentiation and muscle regeneration. Cell Res..

[30] Zhou L., Sun K., Zhao Y., Zhang S., Wang X., Li Y., Lu L., Chen X., Chen F., Bao X. (2015). *Linc-YY1* promotes myogenic differentiation and muscle regeneration through an interaction with the transcription factor YY1. Nat. Commun..

[31] Han X., Yang F., Cao H., Liang Z. (2015). *Malat1* regulates serum response factor through miR-133 as a competing endogenous RNA in myogenesis. FASEB J..

[32] Chen X., He L., Zhao Y., Li Y., Zhang S., Sun K., So K., Chen F., Zhou L., Lu L. (2017). *Malat1* regulates myogenic differentiation and muscle regeneration through modulating MyoD transcriptional activity. Cell Discov..

[33] Wang S., Zuo H., Jin J., Lv W., Xu Z., Fan Y., Zhang J., Zuo B. (2019). Long noncoding RNA *Neat1* modulates myogenesis by recruiting Ezh2. Cell Death Dis..

[34] Hitachi K., Nakatani M., Takasaki A., Ouchi Y., Uezumi A., Ageta H., Inagaki H., Kurahashi H., Tsuchida K. (2019). *Myogenin* promoter-associated lncRNA *Myoparr* is essential for myogenic differentiation. EMBO Rep..

[35] Hitachi K., Nakatani M., Tsuchida K. (2019). Long non-coding RNA *Myoparr* regulates GDF5 expression in denervated mouse skeletal muscle. Noncoding RNA.

[36] Hitachi K., Tsuchida K. (2017). Myostatin-deficiency in mice increases global gene expression at the Dlk1-Dio3 locus in the skeletal muscle. Oncotarget.

[37] Liu M., Li B., Peng W., Ma Y., Huang Y., Lan X., Lei C., Qi X., Liu G.E., Chen H. (2019). LncRNA-MEG3 promotes bovine myoblast differentiation by sponging miR-135. J. Cell. Physiol..

[38] Kallen A.N., Zhou X.B., Xu J., Qiao C., Ma J., Yan L., Lu L., Liu C., Yi J.S., Zhang H. (2013). The imprinted H19 lncRNA antagonizes Let-7 microRNAs. Mol. Cell.

[39] Giovarelli M., Bucci G., Ramos A., Bordo D., Wilusz C.J., Chen C.Y., Puppo M., Briata P., Gherzi R. (2014). H19 long noncoding RNA controls the mRNA decay promoting function of KSRP. Proc. Natl. Acad. Sci. USA.

[40] Dey B.K., Pfeifer K., Dutta A. (2014). The *H19* long noncoding RNA gives rise to microRNAs miR-675-3p and miR-675-5p to promote skeletal muscle differentiation and regeneration. Genes Dev..

[41] Martinet C., Monnier P., Louault Y., Benard M., Gabory A., Dandolo L. (2016). *H19* controls reactivation of the imprinted gene network during muscle regeneration. Development.

[42] Schakman O., Kalista S., Barbé C., Loumaye A., Thissen J.P. (2013). Glucocorticoid-induced skeletal muscle atrophy. Int. J. Biochem. Cell Biol..

[43] Gorjao R., Santos dos C.M.M., Serdan T.D.A., Diniz V.L.S., Alba-Loureiro T.C., Cury-Boaventura M.F., Hatanaka E., Levada-Pires A.C., Sato F.T., Pithon-Curi T.C. (2019). New insights on the regulation of cancer cachexia by N-3 polyunsaturated fatty acids. Pharmacol. Ther..

[44] Soda K., Kawakami M., Kashii A., Miyata M. (1994). Characterization of mice bearing subclones of colon 26 adenocarcinoma disqualifies interleukin-6 as the sole inducer of cachexia. Jpn. J. Cancer Res..

[45] McPherron A.C., Lawler A.M., Lee S.J. (1997). Regulation of skeletal muscle mass in mice by a new TGF-β superfamily member. Nature.

[46] Matsakas A., Mouisel E., Amthor H., Patel K. (2010). Myostatin knockout mice increase oxidative muscle phenotype as an adaptive response to exercise. J. Muscle Res. Cell Motil..

[47] Watts R., Johnsen V.L., Shearer J., Hittel D.S. (2013). Myostatin-induced inhibition of the long noncoding RNA Malat1 is associated with decreased myogenesis. Am. J. Physiol. Cell Physiol..

[48] Alessio E., Buson L., Chemello F., Peggion C., Grespi F., Martini P., Massimino M.L., Pacchioni B., Millino C., Romualdi C. (2019). Single cell analysis reveals the involvement of the long non-coding RNA Pvt1 in the modulation of muscle atrophy and mitochondrial network. Nucleic Acids Res..

[49] Zhang Z.K., Li J., Guan D., Liang C., Zhuo Z., Liu J., Lu A., Zhang G., Zhang B.T. (2018). Long noncoding RNA lncMUMA reverses established skeletal muscle atrophy following mechanical unloading. Mol. Ther..

[50] Sun L., Si M., Liu X., Choi J.M., Wang Y., Thomas S.S., Peng H., Hu Z. (2018). Long-noncoding RNA Atrolnc-1 promotes muscle wasting in mice with chronic kidney disease. J. Cachexia Sarcopenia Muscle.

[51] Zhang Z.K., Li J., Guan D., Liang C., Zhuo Z., Liu J., Lu A., Zhang G., Zhang B.T. (2018). A newly identified lncRNA MAR1 acts as a miR-487b sponge to promote skeletal muscle differentiation and regeneration. J. Cachexia Sarcopenia Muscle.

[52] Neppl R.L., Wu C.L., Walsh K. (2017). lncRNA Chronos is an aging-induced inhibitor of muscle hypertrophy. J. Cell Biol..

[53] Sartori R., Schirwis E., Blaauw B., Bortolanza S., Zhao J., Enzo E., Stantzou A., Mouisel E., Toniolo L., Ferry A. (2013). BMP signaling controls muscle mass. Nat. Genet..

[54] Winbanks C.E., Chen J.L., Qian H., Liu Y., Bernardo B.C., Beyer C., Watt K.I., Thomson R.E., Connor T., Turner B.J. (2013). The bone morphogenetic protein axis is a positive regulator of skeletal muscle mass. J Cell Biol..

[55] Liang T., Zhou B., Shi L., Wang H., Chu Q., Xu F., Li Y., Chen R., Shen C., Schinckel A.P. (2018). lncRNA *AK017368* promotes proliferation and suppresses differentiation of myoblasts in skeletal muscle development by attenuating the function of miR-30c. FASEB J..

[56] Ballarino M., Cipriano A., Tita R., Santini T., Desideri F., Morlando M., Colantoni A., Carrieri C., Nicoletti C., Musaro A. (2018). Deficiency in the nuclear long noncoding RNA *Charme* causes myogenic defects and heart remodeling in mice. EMBO J..

[57] Zhu M., Liu J., Xiao J., Yang L., Cai M., Shen H., Chen X., Ma Y., Hu S., Wang Z. (2017). Lnc-mg is a long non-coding RNA that promotes myogenesis. Nat. Commun..

[58] Eftimie R., Brenner H.R., Buonanno A. (1991). Myogenin and MyoD join a family of skeletal muscle genes regulated by electrical activity. Proc. Natl. Acad. Sci. USA.

[59] Wheeler M.T., Snyder E.C., Patterson M.N., Swoap S.J. (1999). An E-box within the MHC IIB gene is bound by MyoD and is required for gene expression in fast muscle. Am. J. Physiol..

[60] Borensztein M., Monnier P., Court F., Louault Y., Ripoche M.A., Tiret L., Yao Z., Tapscott S.J., Forné T., Montarras D. (2013). Myod and H19-Igf2 locus interactions are required for diaphragm formation in the mouse. Development.

[61] Baruffaldi F., Montarras D., Basile V., De Feo L., Badodi S., Ganassi M., Battini R., Nicoletti C., Imbriano C., Musaro A. (2017). Dynamic phosphorylation of the myocyte enhancer factor 2Cα1 splice variant promotes skeletal muscle regeneration and hypertrophy. Stem Cells.

[62] Shen H., McElhinny A.S., Cao Y., Gao P., Liu J., Bronson R., Griffin J.D., Wu L. (2006). The Notch coactivator, MAML1, functions as a novel coactivator for MEF2C-mediated transcription and is required for normal myogenesis. Genes Dev..

[63] Regué L., Ji F., Flicker D., Kramer D., Pierce W., Davidoff T., Widrick J.J., Houstis N., Minichiello L., Dai N. (2019). IMP2 increases mouse skeletal muscle mass and voluntary activity by enhancing autocrine insulin-like growth factor 2 production and optimizing muscle metabolism. Mol. Cell. Biol..

[64] Koohmaraie M., Shackelford S.D., Wheeler T.L., Lonergan S.M., Doumit M.E. (1995). A muscle hypertrophy condition in lamb (callipyge): Characterization of effects on muscle growth and meat quality traits. J. Anim. Sci..

[65] Davis E., Jensen C.H., Schroder H.D., Farnir F., Shay-Hadfield T., Kliem A., Cockett N., Georges M., Charlier C. (2004). Ectopic expression of DLK1 protein in skeletal muscle of padumnal heterozygotes causes the callipyge phenotype. Curr. Biol..

[66] Gao Y.Q., Chen X., Wang P., Lu L., Zhao W., Chen C., Chen C.P., Tao T., Sun J., Zheng Y.Y. (2015). Regulation of DLK1 by the maternally expressed MIR-379/MIR-544 cluster may underlie callipyge polar overdominance inheritance. Proc. Natl. Acad. Sci. USA.

[67] Zhou Y., Cheunsuchon P., Nakayama Y., Lawlor M.W., Zhong Y., Rice K.A., Zhang L., Zhang X., Gordon F.E., Lidov H.G.W. (2010). Activation of paternally expressed genes and perinatal death caused by deletion of the *Gtl2* gene. Development.

[68] Kota S.K., Llères D., Bouschet T., Hirasawa R., Marchand A., Begon-Pescia C., Sanli I., Arnaud P., Journot L., Girardot M. (2014). ICR noncoding RNA expression controls imprinting and DNA replication at the *Dlk1-Dio3* domain. Dev. Cell.

[69] Butchart L.C., Fox A., Shavlakadze T., Grounds M.D. (2016). The long and short of non-coding RNAs during post-natal growth and differentiation of skeletal muscles: Focus on lncRNA and miRNAs. Differentiation.

[70] Mikovic J., Sadler K., Butchart L., Voisin S., Gerlinger-Romero F., Della Gatta P., Grounds M.D., Lamon S. (2018). MicroRNA and long non-coding RNA regulation in skeletal muscle from growth to old age shows striking dysregulation of the callipyge locus. Front. Genet..

[71] Yu X., Wang Z., Sun H., Yang Y., Li K., Tang Z. (2018). Long non-coding *MEG3* is a marker for skeletal muscle development and meat production traits in pigs. Anim. Genet..

[72] Ciciliot S., Rossi A.C., Dyar K.A., Blaauw B., Schiaffino S. (2013). Muscle type and fiber type specificity in muscle wasting. Int. J. Biochem. Cell Biol..

[73] Amthor H., Macharia R., Navarrete R., Schuelke M., Brown S.C., Otto A., Voit T., Muntoni F., Vrbóva G., Partridge T. (2007). Lack of myostatin results in excessive muscle growth but impaired force generation. Proc. Natl. Acad. Sci. USA.

[74] Honda M., Tsuchimochi H., Hitachi K., Ohno S. (2019). Transcriptional cofactor Vgll2 is required for functional adaptations of skeletal muscle induced by chronic overload. J. Cell. Physiol..

[75] Xing H., Zhou M., Assinck P., Liu N. (2015). Electrical stimulation influences satellite cell differentiation after sciatic nerve crush injury in rats. Muscle Nerve.

[76] He W.A., Berardi E., Cardillo V.M., Acharyya S., Aulino P., Thomas-Ahner J., Wang J., Bloomston M., Muscarella P., Nau P. (2013). NF-κB-mediated Pax7 dysregulation in the muscle microenvironment promotes cancer cachexia. J. Clin. Investig..

[77] Nakanishi R., Hirayama Y., Tanaka M., Maeshige N., Kondo H., Ishihara A., Roy R.R., Fujino H. (2016). Nucleoprotein supplementation enhances the recovery of rat soleus mass with reloading after hindlimb unloading-induced atrophy via myonuclei accretion and increased protein synthesis. Nutr. Res..

[78] Fauconneau B., Paboeuf G. (2000). Effect of fasting and refeeding on in vitro muscle cell proliferation in rainbow trout (Oncorhynchus mykiss). Cell Tissue Res..

[79] Guitart M., Lloreta J., Mañas-Garcia L., Barreiro E. (2018). Muscle regeneration potential and satellite cell activation profile during recovery following hindlimb immobilization in mice. J. Cell. Physiol..

[80] Lee S.J., Huynh T.V., Lee Y.S., Sebald S.M., Wilcox-Adelman S.A., Iwamori N., Lepper C., Matzuk M.M., Fan C.M. (2012). Role of satellite cells versus myofibers in muscle hypertrophy induced by inhibition of the myostatin/activin signaling pathway. Proc. Natl. Acad. Sci. USA.

[81] Russell A.P., Foletta V.C., Snow R.J., Wadley G.D. (2014). Skeletal muscle mitochondria: A major player in exercise, health and disease. Biochim. Biophys. Acta.

[82] Welle S., Cardillo A., Zanche M., Tawil R. (2009). Skeletal muscle gene expression after myostatin knockout in mature mice. Physiol. Genom..

[83] Seene T., Kaasik P., Riso E.M. (2012). Review on aging, unloading and reloading: Changes in skeletal muscle quantity and quality. Arch. Gerontol. Geriatr..

[84] Caron A.Z., Drouin G., Desrosiers J., Trensz F., Grenier G. (2009). A novel hindlimb immobilization procedure for studying skeletal muscle atrophy and recovery in mouse. J. Appl. Physiol..

[85] Batt J.A.E., Bain J.R. (2013). Tibial nerve transection—A standardized model for denervation-induced skeletal muscle atrophy in mice. J. Vis. Exp..

[86] Bhattacharya A., Hamilton R., Jernigan A., Zhang Y., Sabia M., Rahman M.M., Li Y., Wei R., Chaudhuri A., Van Remmen H. (2014). Genetic ablation of 12/15-lipoxygenase but not 5-lipoxygenase protects against denervation-induced muscle atrophy. Free Radic. Biol. Med..

[87] Furuya N., Ikeda S.I., Sato S., Soma S., Ezaki J., Oliva Trejo J.A., Takeda-Ezaki M., Fujimura T., Arikawa-Hirasawa E., Tada N. (2014). PARK2/Parkin-mediated mitochondrial clearance contributes to proteasome activation during slow-twitch muscle atrophy via NFE2L1 nuclear translocation. Autophagy.

[88] Pigna E., Renzini A., Greco E., Simonazzi E., Fulle S., Mancinelli R., Moresi V., Adamo S. (2018). HDAC4 preserves skeletal muscle structure following long-term denervation by mediating distinct cellular responses. Skelet. Muscle.

[89] Michel R.N., Cowper G., Chi M.M., Manchester J.K., Falter H., Lowry O.H. (1994). Effects of tetrodotoxin-induced neural inactivation on single muscle fiber metabolic enzymes. Am. J. Physiol..

[90] Liu H., Thompson L.V. (2019). Skeletal muscle denervation investigations: Selecting an experimental control wisely. Am. J. Physiol. Cell Physiol..

[91] Kamei Y., Miura S., Suzuki M., Kai Y., Mizukami J., Taniguchi T., Mochida K., Hata T., Matsuda J., Aburatani H. (2004). Skeletal muscle FOXO1 (FKHR) transgenic mice have less skeletal muscle mass, down-regulated Type I (slow twitch/red muscle) fiber genes, and impaired glycemic control. J. Biol. Chem..

[92] Yakabe M., Ogawa S., Ota H., Iijima K., Eto M., Ouchi Y., Akishita M. (2018). Inhibition of interleukin-6 decreases atrogene expression and ameliorates tail suspension-induced skeletal muscle atrophy. PLoS ONE.

[93] Wada S., Kato Y., Okutsu M., Miyaki S., Suzuki K., Yan Z., Schiaffino S., Asahara H., Ushida T., Akimoto T. (2011). Translational suppression of atrophic regulators by microRNA-23a integrates resistance to skeletal muscle atrophy. J. Biol. Chem..

[94] Jesinkey S.R., Korrapati M.C., Rasbach K.A., Beeson C.C., Schnellmann R.G. (2014). Atomoxetine prevents dexamethasone-induced skeletal muscle atrophy in mice. J. Pharmacol. Exp. Ther..

[95] Gomes M.D., Lecker S.H., Jagoe R.T., Navon A., Goldberg A.L. (2001). Atrogin-1, a muscle-specific F-box protein highly expressed during muscle atrophy. Proc. Natl. Acad. Sci. USA.

