# Supplementary material for: Expression Levels of Long Non-Coding RNAs Change in Models of Altered Muscle Activity and Muscle Mass
Source: Int J Mol Sci. 2020 Feb 27;21(5):1628. doi: 10.3390/ijms21051628 (PMC7084395; doi:10.3390/ijms21051628)
Supplement: Supplementary file 1 [file ijms-21-01628-s001.zip › ijms-702060 suppl for final2/Supplementary Files.pdf]

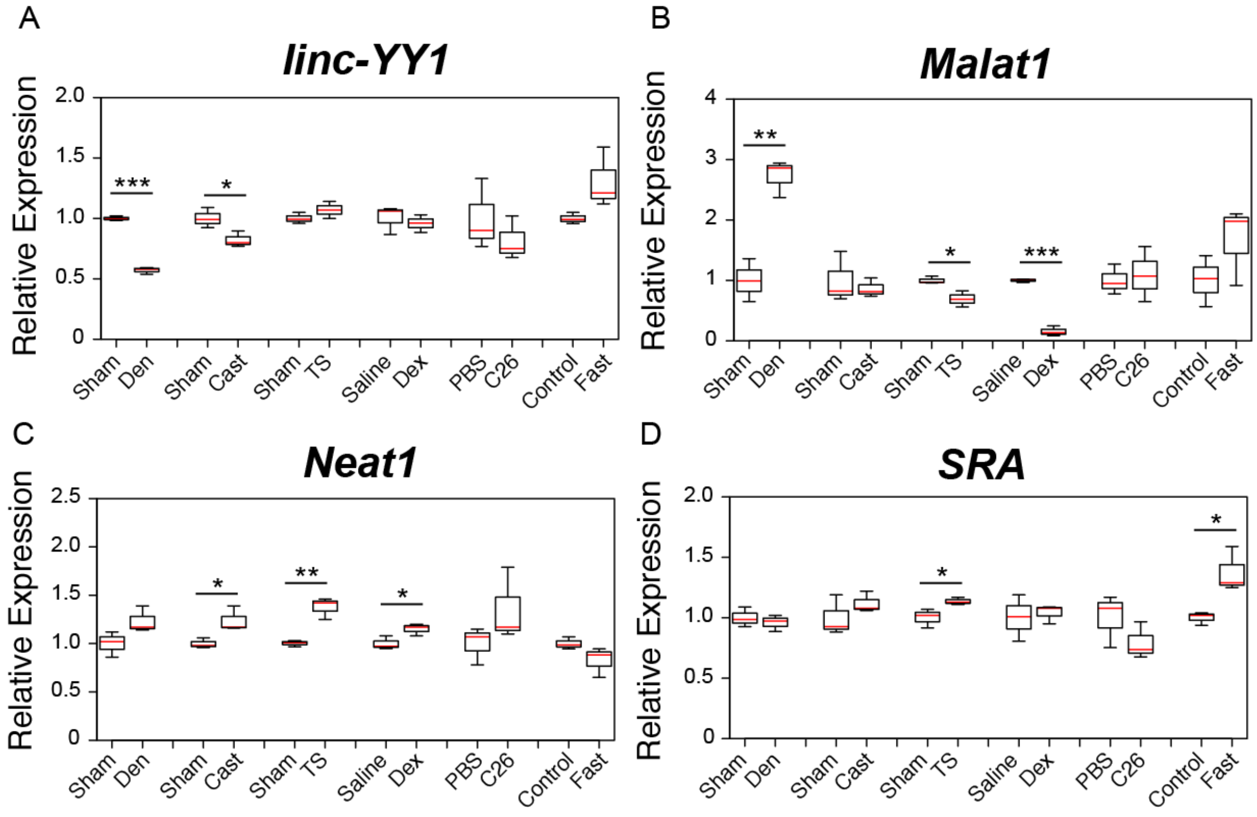

Supplementary Figure S1. Changes in the expression of skeletal muscle differentiation-related lncRNAs in six muscle atrophy conditions. (**A**–**D**) Box-and-whisker plots showing the results of quantitative RT-PCR (qRT-PCR) for *linc-YY1* (**A**), *Malat1* (**B**), *Neat1* (**C**), and *SRA* (**D**) expression in multiple muscle atrophy models. Sham; tibialis anterior (TA) muscles of sham-operated C57BL/6J mice. Den; denervated TA muscles of C57BL/6J mice. Cast; casting-operated TA muscles of C57BL/6J mice. TS; TA muscles of tail suspension-operated C57BL/6J mice. Saline; TA muscles of saline-injected C57BL/6J mice. Dex; TA muscles of dexamethasone-injected C57BL/6J mice. PBS; TA muscles of control phosphate-buffered saline (PBS)-injected CD2F1 mice. C26; TA muscles of C26 tumor-bearing CD2F1 mice. Control; TA muscles of C57BL/6J mice provided with water and food ad libitum. Fast; TA muscles of fasting C57BL/6J mice. qRT-PCR data were normalized to *Rpl26* expression and shown as relative expression. $n = 3$ per group. * $p < 0.05$. ** $p < 0.01$. *** $p < 0.001$.

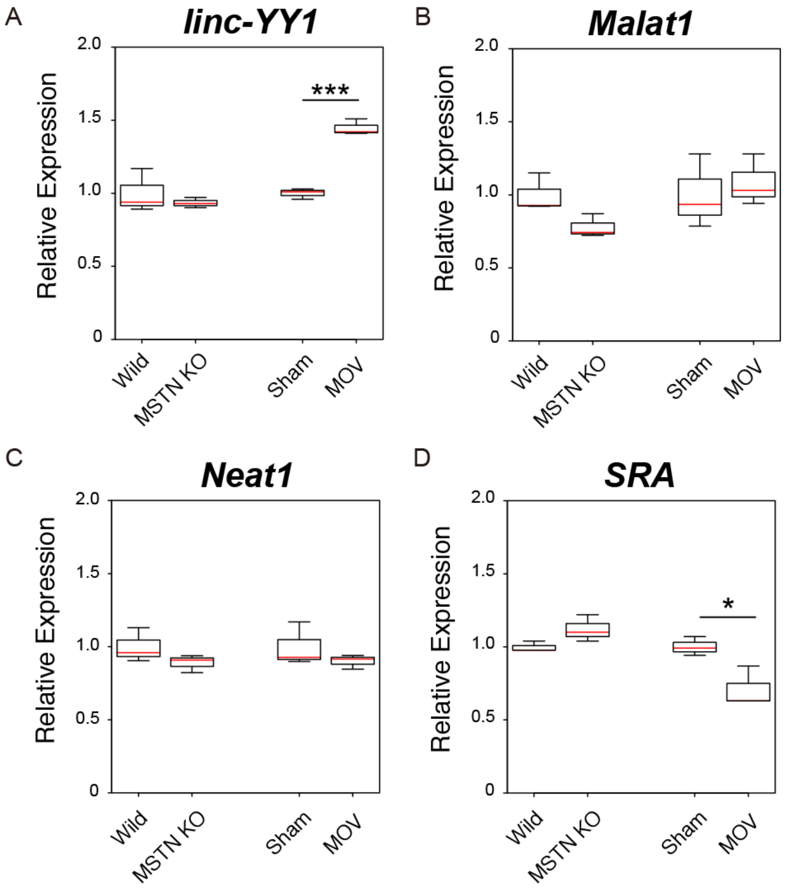

Supplementary Figure S2. Changes in the expression levels of skeletal muscle differentiation-related lncRNAs in skeletal muscle hypertrophy conditions. (**A**–**D**) Box-and-whisker plots showing the results of quantitative RT-PCR (qRT-PCR) for *linc-YY1* (**A**), *Malat1* (**B**), *Neat1* (**C**), and *SRA* (**D**) expression in two muscle hypertrophy conditions. Wild; tibialis anterior (TA) muscles of control C57BL/6J mice. MSTN KO; TA muscles of C57BL/6J-background *myostatin* knockout mice. Sham; plantaris muscles of sham-operated C57BL/6J mice. MOV; plantaris muscles of mechanical overload (MOV)-operated C57BL/6J mice. qRT-PCR data were normalized to *Rpl26* expression and shown as relative expression. $n = 3$ per group. * $p < 0.05$. *** $p < 0.001$.
